# Unravelling the mystery of female meiotic drive: where we are

**DOI:** 10.1098/rsob.210074

**Published:** 2021-09-01

**Authors:** Frances E. Clark, Takashi Akera

**Affiliations:** Cell and Developmental Biology Center, National Heart, Lung, and Blood Institute, National Institutes of Health, Bethesda, MD, USA

**Keywords:** meiotic drive, female meiosis, chromosome segregation, selfish genetic elements

## Abstract

Female meiotic drive is the phenomenon where a selfish genetic element alters chromosome segregation during female meiosis to segregate to the egg and transmit to the next generation more frequently than Mendelian expectation. While several examples of female meiotic drive have been known for many decades, a molecular understanding of the underlying mechanisms has been elusive. Recent advances in this area in several model species prompts a comparative re-examination of these drive systems. In this review, we compare female meiotic drive of several animal and plant species, highlighting pertinent similarities.

## Graphical abstract

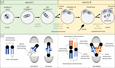

## 1. Introduction

Genetic conflict exists in many forms and has been credited with impacting gene expression, genome evolution and speciation [1]. Broadly, genetic conflict can be categorized as either interindividual or intraindividual [2]. Interindividual genetic conflict involves genes in different individuals, such as parents and offspring who have conflicting optima for parental resource allocation. Intraindividual genetic conflict typically occurs between genetic elements within the same individual, but with different patterns of inheritance, such as nuclear and mitochondrial genomes [2]. Yet even different portions of the nuclear genome can experience conflict with each other if one selfishly alters its own pattern of inheritance. The process by which a genetic element increases its own transmission above that expected by Mendel's law of segregation is known as drive [3]. There is a multitude of strategies by which drive can be accomplished, all of which fall into one of three categories: interference (reducing transmission of competitors, e.g. selfish mitochondria), overreplication (replicating more frequently than once per mitosis or meiosis, e.g. transposable elements) or gonotaxis (segregating towards the germline, e.g. B chromosomes) [3]. Drive enables a selfish genetic element to increase in frequency, even if that element, or linked alleles, decrease fitness. Indeed, loci that experience drive often incur a reduction in fitness and are thought to engage in an evolutionary arms race with the rest of the genome as the genome evolves mechanisms to suppress the drive [2,4–8]. However, it is important to note that while the process of drive is a selfish one, loci that experience drive can also be neutral, or even beneficial to an organism [2,5].

**Figure 1.**
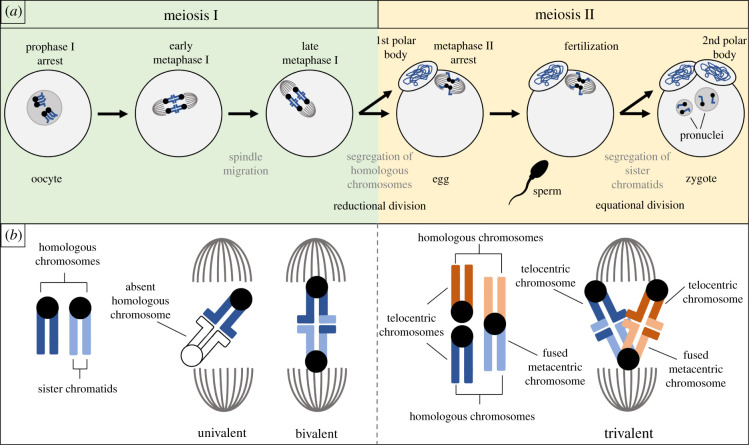
Female meiosis in animals. (*a*) Typical progression through animal oogenesis is depicted for a cell with two chromosome pairs (*n* = 2). (*b*) Homologous chromosomes can remain unpaired (univalent) or pair (form a bivalent or trivalent) during meiosis I.

Gametogenesis is a critical timepoint for selfish genetic elements to achieve drive [3]. Meiotic drive, a term introduced in 1957 by Sandler & Novitski [9], refers to drive that occurs during gametogenesis. When and how meiotic drive transpires depends on the sex in which it occurs. Male meiotic drive is an example of interference that takes advantage of the natural competition between sperm cells [7]. Not all sperm will fertilize an egg, so they are in direct competition with each other, racing towards an egg. As one might expect from a selfish ‘cheater’ trying to win a race, male meiotic drive occurs when a genetic element confers the ability to sabotage competitor sperm cells, even though those sperm cells were produced by the same male. A similar drive is also observed in yeast meiosis where the selfish genetic element is commonly referred to as a spore killer [10–12]. These drive systems are well characterized in previous papers [7,13–15] and will not be discussed further in this review. Female meiotic drive is an example of gonotaxis that occurs *during* meiosis, not after, and does not require the killing of female gametes [7,16,17]. Unlike spermatogenesis, oogenesis results in a single gamete (i.e. egg) per meiotic event (figure 1*a*). The other, non-gamete products of oogenesis are known as polar bodies and do not have an opportunity to contribute to the next generation, but instead often disintegrate. This means an allele in a genome about to undergo female meiosis will either be segregated to the egg (where it will be transmitted) or to the polar body (an evolutionary dead end). Female meiotic drive occurs when a genetic element increases the likelihood that it will be segregated to the egg and avoid the polar body. This preferential segregation will be the focus of this review.

In animals, oogenesis begins before birth and is paused in meiosis I during prophase. After reaching sexual maturity, a number of oocytes resume meiosis with each menstrual cycle. Homologous chromosomes will separate at anaphase of meiosis I, then one set of chromosomes will be extruded in the first polar body while the other set is retained in the egg, and meiosis will pause for a second time at metaphase of meiosis II. Meiosis II is only completed if fertilization occurs. Following fertilization, sister chromatids will separate at anaphase of meiosis II and the second polar body will be extruded (figure 1*a*). It is perhaps more intuitive to consider female meiotic drive that occurs during meiosis I, as this is when homologous chromosomes segregate. However, as will be discussed in this review, meiotic drive can also occur in meiosis II. Both times that chromosomes are segregated and extruded with a polar body represent an opportunity for selfish genetic elements to manipulate cellular mechanisms in order to avoid the polar body. In plants, meiosis and fertilization are separated by what is known as a haploid generation. While haploidy in animals is a highly transitory state and fertilization (which will return the cell to diploidy) occurs before any mitoses, plants spend a larger portion of their life cycle as haploids. Meiosis in a sporophyte produces single-celled, haploid spores (microspores from males and megaspores from females). Spores undergo mitotic divisions generating a multicellular gametophyte which is entirely haploid. The gametophyte partitions gametes (eggs and sperm) which will eventually meet in fertilization producing a single-celled diploid zygote. This zygote will undergo mitotic divisions and develop into a multicellular diploid sporophyte, and so the cycle continues. Despite the difference in the timing of fertilization, female meiosis in plants will segregate homologous chromosomes in meiosis I and segregate sister chromatids in meiosis II, and is asymmetrical as it results in only one viable haploid cell, just like animal female meiosis. For this reason, female meiotic drive can occur in animals as well as plants.

Female meiotic drive is dependent upon three conditions [18]. The first is the asymmetry in cell fate discussed above. The production of polar bodies and the fact that only one sister chromatid of four from a pair of homologous chromosomes will make it into the egg provides the opportunity for there to be one ‘winner’ and three ‘losers’ (figure 1*a,b*). Of course, from a genetic and evolutionary standpoint, which chromatid becomes the ‘winner’ is irrelevant if all four chromatids are identical. For this reason, the second condition required for female meiotic drive is heterozygosity [18]. Typically, heterozygosity invokes the idea of the ‘Aa genotype’, or an individual with two different alleles for the same gene on a pair of homologous chromosomes. However, as we will discuss below, monosomic chromosomes that form univalents (unpaired chromosomes) in meiosis also meet the requirement of heterozygosity. Here the genotype can be thought of as the ‘AO genotype’ where ‘O’ signifies the lack of a second allele or chromosome. The reason the absence of a second chromosome is just as important as the presence of one is that the products of meiosis could potentially have (representing the ‘winning’ situation) or not have (representing the ‘losing’ situation) this chromosome. And finally, the third condition is asymmetry in cell structure, typically thought to be asymmetry within the meiotic spindle [18]. Essentially, some asymmetric structure must distinguish which side of the metaphase plate will give rise to the egg, and which will give rise to the polar body. Imagine standing in a hallway with two doors on either side; you know one door will lead you somewhere nice, while the other door will lead you somewhere decidedly less pleasant. You can make the decision that you want to enter the ‘somewhere nice’ door, but without any means of distinguishing which door is which, you are left with a Mendelian 50 : 50 chance.

Asymmetry in cell fate has long been established [19]. It is known to have evolved multiple times and to be a conserved feature in female meiosis in animals as well as in spermatophytes [20]. Asymmetry in positioning of the meiotic spindle is also well characterized. Shortly after meiosis I resumes in females, the spindle migrates towards the cortex and assumes a perpendicular orientation to the cortex (figure 1*a*). This feature is highly conserved among animals and known to facilitate polar body formation [21]. However, the side of the spindle that migrates towards the cortex is thought to be random, and the question remains, how can a chromosome interact with the spindle to preferentially segregate to the egg? Furthermore, a clear, molecular understanding of the functional differences in loci that experience drive when heterozygous and asymmetric structures which allow drive to occur has been far more difficult to achieve. This review will touch on various types of female meiotic drive observed in different plant and animal species, with the aim of drawing comparisons across these examples in the context of recent, significant advancements.

## 2. Drive involving bivalents and trivalents

### 2.1. Competition between homologues

**Figure 2.**
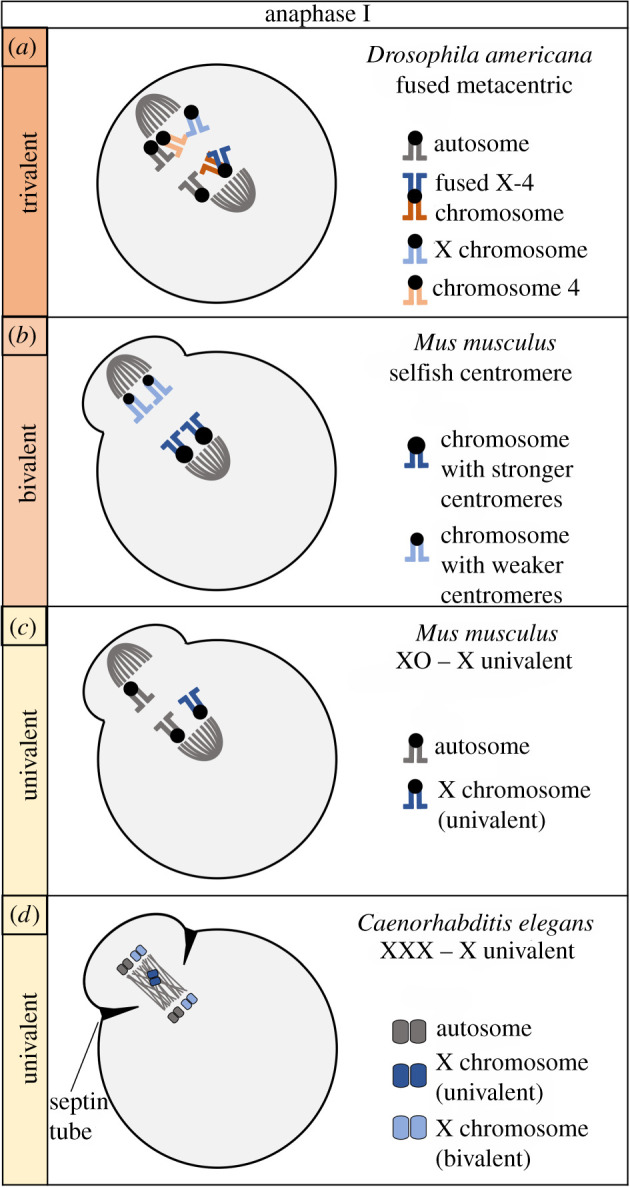
Comparison of female meiotic drive systems. Meiosis I is depicted for four examples of female meiotic drive in three animal species. Drive involving trivalents in *Drosophila* (*a*) and bivalents in mice (*b*) may be mechanistically distinct from the drive of univalent chromosomes in mice (*c*) and worms (*d*).

Several comprehensive studies have investigated preferential segregation involving a bivalent or trivalent (figures 1*b* and 2*c,d*). In bivalents, heterozygosity can exist at centromeric or non-centromeric loci, leading to biased segregation. Meiotic drive of centromeric loci is often called centromere drive (figure 2*b*) [22]. Centromere drive is thought to be responsible for the remarkable lack of conservation of DNA sequences and proteins required for centromere and kinetochore function [23,24]. Additionally, in individuals heterozygous for chromosome fusions or fissions, preferential segregation involving a trivalent is believed to be responsible for rapid karyotype evolution and the propensity for a species' karyotype to be mostly telocentric or mostly metacentric (figure 2*a*) [16]. In their 2001 study, Pardo-Manuel de Villena & Sapienza [16] proposed the ‘unequal centromere number rule’, which attributed the preferential segregation observed with trivalents (and even univalents) to the difference in number of distinct centromeres on either side of the spindle. While acknowledging that the molecular mechanisms were entirely unknown, they speculated that the side of the spindle that was more efficient or faster with respect to capturing centromeres would be more likely to capture the greater number of centromeres, leading to preferential segregation. Importantly, the direction of preferential segregation is not consistent from one species to the next [16].

Similar in nature to the concept of spindle asymmetry in centromere attachment efficiency, centromere drive was suggested to involve centromere asymmetry, where ‘stronger’ centromeres build bigger kinetochores and attach to the spindle more efficiently than the paired ‘weaker’ centromere [23]. Over a decade later, a succession of studies greatly improved our understanding of the preferential segregation involving a trivalent as well as centromere drive in bivalents, and it appears that the two are mechanistically linked.

### 2.2. Chromosome fusions experience drive controlled by kinetochore size

Robertsonian fusions are metacentric chromosomes formed when two telocentric chromosomes fuse at their centromeres (figure 1*b*). They produce trivalents in meiosis I in heterozygous individuals when the fused chromosome pairs with the two unfused homologues (figure 1*b*). In at least one species of *Drosophila*, fused metacentric chromosomes preferentially segregate to the egg (figure 2*a*). *Drosophila americana* has acquired two metacentric chromosomes, one when the 2nd and 3rd chromosomes fused and another when the X and 4th chromosomes fused [25]. The X–4 metacentric is transmitted to approximately 57% of offspring in both intra- and interspecific hybrids (drive in intraspecific hybrids is depicted in figure 2*a*). The 2–3 metacentric is transmitted to approximately 63% of offspring in interspecific hybrids (table 1). Importantly, the authors examined three paracentric inversions, none of which altered the strength of drive, suggesting meiotic drive is controlled by the centromere, and not inversion polymorphisms [25].

**Table 1.** Overview of drive systems. This table provides a list of drive systems discussed in this review. Frequency of orientation or segregation towards the egg pole and the references that provided these data are provided. These frequencies were used to calculate a ratio indicating how often the selfish element orients/segregates towards the egg or the polar body. Rows are organized by whether univalents, bivalents or trivalents are observed in meiosis I. *Triploid female oysters were shown to produce 12% aneuploid offspring when mated to a diploid male [26]. This 12% was excluded from consideration for table 1 and the ratio of diploid and triploid offspring were used to calculate the ratio of preferential segregation.

species name	genotype of driving element	orientation or segregation toward egg	ratio	reference	
*Mus musculus*	XO	60%	1.5 : 1	LeMaire-Adkins *et al*. [27]; LeMaire-Adkins & Hunt [28]	univalent
*Caenorhabditis elegans*	XXX	29%	1 : 2.4	Cortes *et al*. [29]	
*Crassostrea gigas*	triploid	35%*	1 : 1.9	Gong *et al*. [26]	bivalent
*Mus musculus*	heterozygous at centromeres	62%	1.6 : 1	Iwata-Otsubo *et al*. [30]	
*Mimulus guttatus*	heterozygous for D allele	58–100%	1.4 : 1	Fishman & Kelly [31]	
*Zea mays*	heterozygous for Ab10	83%	4.9 : 1	Buckler *et al*. [32]	
*Mus musculus*	heterozygous for HSR	85%	5.7 : 1	Agulnik *et al*. [33]	
*Mus musculus*	heterozygous for R2d2	95%	19 : 1	Didion *et al*. [34]	
*Mus musculus*	heterozygous for Om	56%	1.3 : 1	Wu *et al*. [35]	
*Drosophila americana*	heterozygous for metacentric fusion	57–63%	1.3–1.7 : 1	Stewart *et al*. [25]	trivalent
*Mus musculus*	heterozygous for metacentric fusion	40%	1 : 1.5	Chmátal *et al*. [37]	

*Mus musculus domesticus* mice heterozygous for Robertsonian fusions experience transmission bias in favour of the two unfused telocentric chromosomes of each trivalent [16]. Using mice heterozygous for Rb(6.16), Rb(2.17) or Rb(7.18), Chmátal *et al*. found asymmetry in centromere and kinetochore protein localization with the telocentric chromosomes, which preferentially segregate to the egg, recruiting more kinetochore proteins than the metacentric chromosome [37]. This is significant for several reasons. First, it suggests preferential segregation involving a trivalent is not due to the number of centromeres, but the ability of each centromere to recruit kinetochore proteins. This could mean that the mechanism of drive could be quite similar between trivalents and centromere drive in bivalents, where the ‘stronger’ centromere is proposed to build a bigger kinetochore. If so, it would be interesting to test if the X–4 and 2–3 metacentrics in *D. americana* have relatively larger kinetochores. Second, it explains why the direction of biased segregation differs from one species to the next. If there is variation among species, or populations, in the size of kinetochores, then species that build smaller kinetochores on telocentrics (and therefore have ‘weaker’ centromeres compared to metacentrics) are likely to have Robertsonian fusions which have preferentially fixed in the population. Therefore, these species are expected to have mostly metacentric karyotypes [37]. Conversely, species with stronger centromeres that build bigger kinetochores on telocentrics compared to metacentrics will probably preferentially segregate Robertsonian fusions to the polar body and have mostly telocentric karyotypes. This raises the question, why do telocentric chromosomes with large kinetochores form metacentric chromosomes with relatively smaller kinetochores after a chromosome fusion? One hypothesis is that there is a constraint on kinetochore size, and that exceeding the upper size limit results in fitness costs (e.g. errors in mitosis and meiosis), creating a selective force that eliminates fusions except those that decrease kinetochore size, in species with bigger kinetochores [36,38].

**Figure 3.**
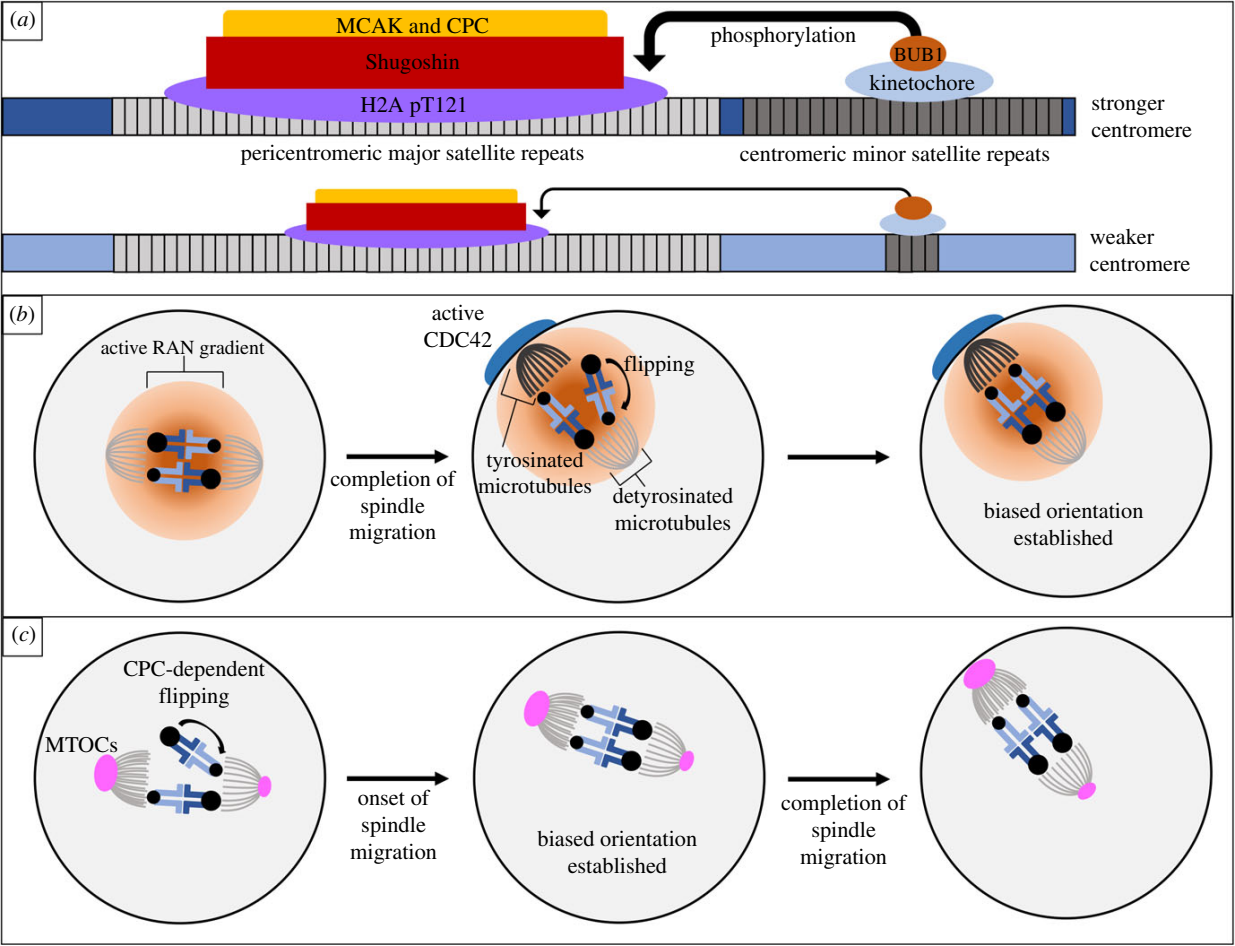
Molecular mechanisms of centromere drive in mice. (*a*) Stronger centromeres build larger kinetochores which recruit more destabilizers compared to weaker centromeres in mice. (*b*) In addition to this centromere asymmetry, cortical positioning of the spindle induces spindle asymmetry in microtubule tyrosination, facilitating directional flipping until stronger centromeres are preferentially oriented in late metaphase I. (*c*) In a *Mus musculus* hybrid, heterozygous for chromosome 4 and 17 centromere size, studied by Wu *et al.* [39], asymmetry is seen in microtubule and MTOC density. In this system, larger centromeres preferentially orient prior to the completion of spindle migration.

To understand how centromeres can regulate kinetochore protein recruitment such that different centromeres build bigger or smaller kinetochores, a 2017 study searched for genetic or epigenetic differences between stronger and weaker centromeres [30]. Intraspecific crosses were made between *Mus musculus domesticus* stronger centromere strains and weaker centromere strains. In bivalents of these intraspecific hybrids, the stronger centromeres recruit more kinetochore proteins than the weaker centromeres (figure 3*a*). Bivalents of these hybrids preferentially oriented stronger centromeres towards the egg pole 62% of the time (figure 2*b*). Importantly, the authors found that the stronger centromeres contained 6–10× more minor satellite, a centromeric repeat in mice, than the weaker centromeres. These results implicate centromere repeat expansion as being at least partially responsible for the recruitment of more kinetochore proteins (see below) [30].

### 2.3. Asymmetric kinetochores interact with the asymmetric spindle

While functional heterozygosity, like stronger and weaker centromeres, is required for meiotic drive, so is an asymmetry in cell structure [18]. To understand how stronger, larger kinetochores could interact with the spindle such that biased segregation could be achieved, another 2017 study looked for asymmetry in the microtubules (which interact with kinetochores) of either side of the spindle [40]. Looking at post-translational modifications of tubulins, they found asymmetry in the amount of tyrosinated and detyrosinated α-tubulin, with more tyrosinated α-tubulin on the cortical side of the spindle. This asymmetry occurred once the spindle had positioned itself close to the cortex in late metaphase I, but not before. It was previously established that as the spindle migrates to the cortex, a chromatin-based RAN activity gradient causes cortical polarization and enriches active CDC42 (figure 3*b*) [41–45]. Experiments with constitutive-active and dominant-negative mutants of RAN, as well as CDC42, revealed that without cortical polarization, spindle asymmetry was lost. Furthermore, this spindle asymmetry was essential for the stronger centromeres to preferentially orient towards the egg pole shortly before anaphase I. This suggests that as the centrally located symmetric spindle migrates towards the cortex, cortical CDC42 signals increase tyrosinated α-tubulin on the cortical side of the spindle, which serves as a spatial cue for selfish centromeres to distinguish the egg and cortical pole (figure 3*b*). Because centromeres form microtubule attachments prior to spindle migration and the establishment of the necessary spindle asymmetry, the authors examined re-orientation or flipping of bivalents on the spindle. They found that stronger centromeres, especially when located on the cortical side of the spindle, had more unstable microtubule attachment and facilitated bivalents to flip after spindle migration (figure 3*b*) [40].

While this finally illuminated the molecular nature of structural asymmetry required for centromere drive, an important question still remains. How do stronger centromeres interact with the spindle with tyrosinated α-tubulin asymmetry in order to produce this biased flipping behaviour and affect orientation towards the egg? Taking an important first step towards addressing this question, a 2019 study was able to show that major microtubule destabilizing factors, mitotic centromere-associated kinesin (MCAK) and the chromosome passenger complex (CPC), were recruited more to the stronger centromeres in intraspecific hybrid mice (figure 3*a*) [38,46–48]. Microtubule destabilizers are recruited to pericentromeres and are critical for error correction during cell division [49,50]. The increase in major destabilizing factors (MCAK and CPC) could explain the increased susceptibility of stronger centromeres to detach from microtubules, particularly tyrosinated microtubules due to their unstable nature compared to detyrosinated microtubules [51,52]. However, how building larger kinetochores leads to the recruitment of more destabilizers at pericentromeres was still mysterious. It was previously shown that kinetochore-localized BUB1 catalyses histone H2A phosphorylation (H2A pT121) to recruit Shugoshin, which is the scaffold for MCAK and CPC at the pericentromeres [53,54]. The authors of the 2019 study found that the entire BUB1 pathway is amplified on stronger centromeres [38]. Experimental recruitment of BUB1 to pericentromeric major satellite cancelled the asymmetry by increasing MCAK and CPC levels across the bivalent, and biased orientation was lost under this condition. Taken together, this suggests that expanded centromeric minor satellite repeats build larger kinetochores, with more BUB1, which in turn recruit more destabilizing factors such as MCAK to the pericentromere, making stronger centromeres more prone to microtubule detachment and flipping. When the spindle migrates towards the cortex, an increase in tyrosinated α-tubulin on the cortical side of the spindle makes detachment more likely to happen on the cortical side, resulting in biased flipping towards the egg side (figure 3*b*) [38]. From differences in centromere DNA sequence to spindle asymmetry, the studies discussed above provide a strong start to a comprehensive molecular model for the mechanism of centromere drive.

In order to confirm that the molecular mechanisms were, at least to some extent, universal, the 2019 study examined centromere drive in an interspecific hybrid between *Mus musculus* and *Mus spretus* [38]. In these hybrids, the *M. spretus* centromeres have more centromeric minor satellite than even the larger *M. musculus* centromeres. Surprisingly, kinetochore proteins and BUB1 were not asymmetrically enriched on the *M. musculus x M. spretus* hybrid bivalents. Interestingly, even without asymmetric kinetochore size, H2A pT121, Shugoshin and MCAK showed asymmetric localization with higher enrichment on *M. spretus* centromeres. The authors showed that while BUB1 quantities were the same across the bivalent, increased localization of condensin II to the *M. spretus* centromeres could induce differences in centromere geometry, which allows BUB1 kinase more access to the *M. spretus* pericentromeres to recruit more destabilizing factors there [38,55]. Much as they say ‘all roads lead to Rome’, the centromeres in these two species (*M. musculus* and *M. spretus)* are both using destabilizing activity to bias their segregation, but each species has evolved a distinct pathway to recruit this activity. This supports the idea that centromere drive is not an isolated, rare event, but a much more frequent form of genetic conflict fuelling the rapid evolution of centromere DNA and proteins as theorized by Henikoff *et al.* [23] and Malik *et al.* [24].

A nice comparison to these studies is a study from 2018 which found that another *Mus musculus domesticus* hybrid, heterozygous for Chromosome 4 and 17 centromere size, experience preferential segregation [39]. In this system, the stronger, larger centromeres, which build slightly smaller kinetochores, preferentially orient towards the egg pole in early metaphase I, prior to the completion of spindle migration (figure 3*c*). Like the previous studies, initial orientation was not biased, but directional flipping prior to spindle migration did result in preferential orientation [39]. The authors showed that inhibiting Aurora B/C kinases, catalytic subunits of CPC, resulted in a loss of re-orientation. As CPC destabilizes microtubule interactions, particularly those experiencing low tension, the authors examined the spindle for asymmetry that CPC could act upon. They found evidence for a greater density of microtubules and microtubule-organizing centres (MTOCs) on what would become the cortical side of the spindle, prior to spindle migration. They also found that intra-kinetochore tension is higher on the cortical side of the spindle during spindle migration. Taken together, this suggests that spindle asymmetry begins prior to spindle migration in this system, when more MTOCs produce more microtubules on one side of the spindle, which results in a greater force exerted on kinetochores facing that pole. This asymmetric pull on bivalents leads CPC to destabilize microtubule interactions, causing bivalents to re-orient [39]. How the side of the spindle with more MTOCs and microtubules migrates to the cortex and how centromere asymmetry and spindle asymmetry interact to accomplish biased flipping remain interesting questions in this system. This study captures another system of drive, in the same taxonomic group, that uses stronger, larger centromeres to preferentially orient, but exploits an earlier asymmetry within the spindle. This emphasizes the multitude of ways in which selfish genetic elements can manipulate standard mechanisms in female meiosis in order to accomplish drive. As more examples are characterized, it will be interesting to evaluate whether or not certain aspects of meiosis are more prone to these manipulations by selfish elements than others. Centromere drive in monkeyflowers (*Mimulus guttatus*) can provide a significant comparison. In monkeyflowers, an expanded repeat on one chromosome called centromere-associated driver, or the D allele, is thought to drive in meiosis I [56]. The D allele is transmitted over the non-D allele 58% of the time in intraspecific crosses, and nearly 100% of the time in interspecific crosses (table 1) [31]. Recent work in this system has focused on characterizing the evolutionary consequences of this drive, and the underlying mechanisms remain unknown [57]. As the molecular mechanisms are characterized, the comparison between centromere drive in mice and plants will provide a broader taxonomic context for our understanding of how female meiosis is manipulated by selfish centromeres.

### 2.4. Role of the pericentromere in centromere drive

In the mouse examples discussed above, stronger centromeres interact with spindle asymmetries by recruiting destabilizing factors to pericentromeres in order to directionally re-orient bivalents and possibly trivalents. Understanding the importance of destabilizing factors has revealed a role for the pericentromere in centromere drive. Destabilizing factors can be recruited by two distinct pathways, the kinetochore pathway and the heterochromatin pathway [58]. The kinetochore pathway (discussed above) functions through kinetochore recruitment of BUB1 [38,53,54]. Independently, the CPC can localize to pericentromeric heterochromatin and recruit SGO2 and MCAK in the heterochromatin pathway [58–61]. Kumon *et al*. suggest that evolutionary shifts which lead to less reliance on the kinetochore pathway and more reliance on the heterochromatin pathway is a route for genome suppression of centromere drive [58]. Conversely, it is possible that centromeric and pericentromeric DNA function selfishly as one unit and manipulate their pericentromere to recruit more destabilizing factors. Experimental manipulation of centromeric and pericentromeric DNA would provide a clearer picture of how each satellite sequence can contribute to centromere drive. The D allele in monkeyflowers is an expanded repeat adjacent to the typical centromere repeats [56]. Studies investigating whether the D allele can function similarly to centromere repeats or pericentromere repeats would provide further insights into these mechanisms.

### 2.5. Non-centromeric meiotic drivers

**Figure 4.**
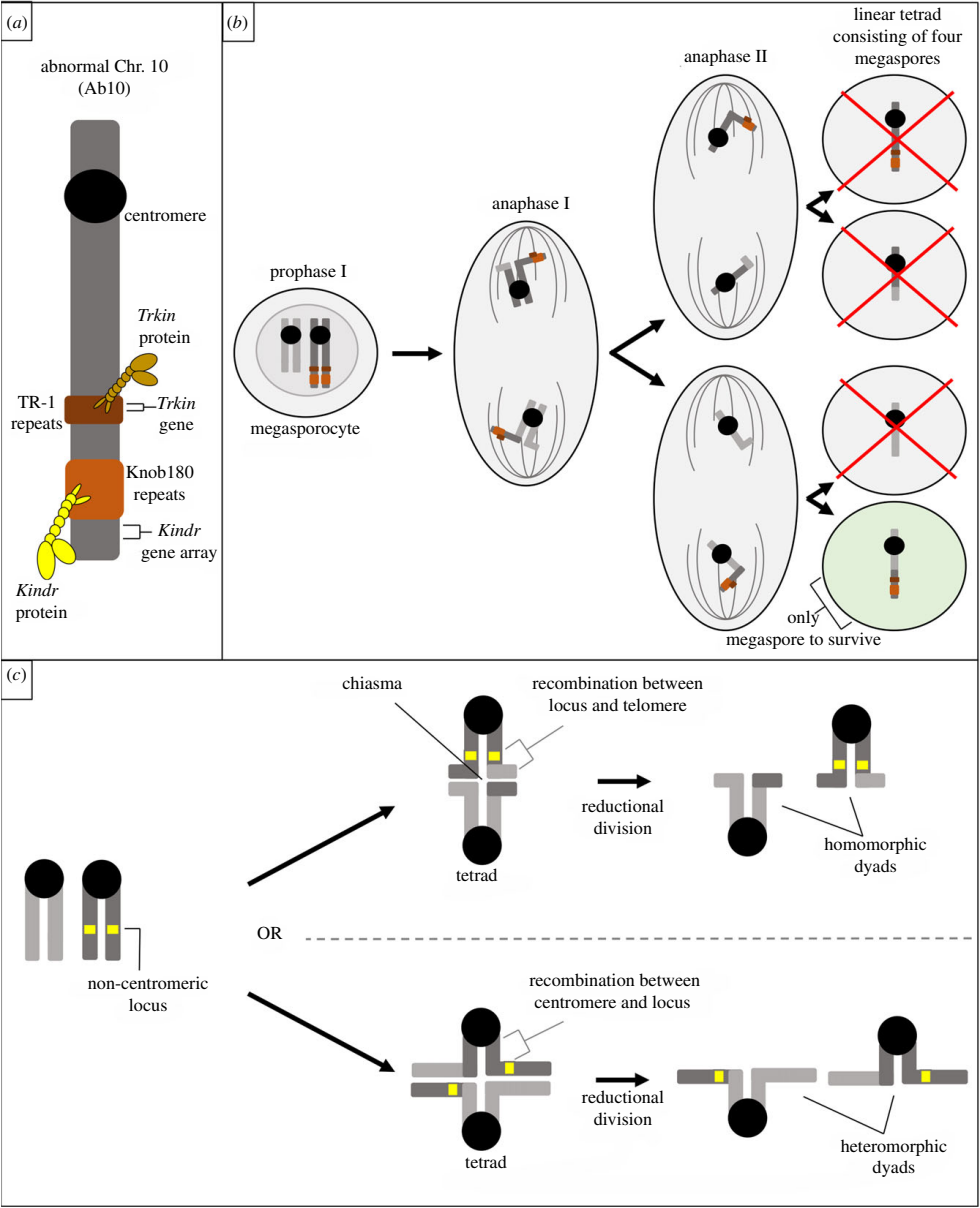
Molecular mechanisms of the drive of knob domains in maize. (*a*) Kinesin-14 motor proteins, *Kindr* and *Trkin*, bind to knob180 and TR-1 repeats, respectively, in meiosis I and II. (*b*) *Kindr* and *Trkin* travel faster on microtubules, pulling the knob towards the upper and lower megaspores. This increases the likelihood that the knob domain will be incorporated into the lower megaspore which will become the egg. (*c*) When chromosomes pair, they recombine and remain attached by chiasmata. If recombination occurs between the heterozygous locus of interest and the telomere, both sister chromatids on the same side of the bivalent have the same allele, resulting in homomorphic dyads. If recombination occurs between the centromere and a heterozygous locus of interest, strands are exchanged such that each side of the bivalent now has one of each allele for that locus. This creates heteromorphic dyads.

Significant advancements have also been made in characterizing meiotic drive of non-centromeric loci, such as knob domains in maize, *Zea mays* (figure 4*a,b*). These heterochromatic knobs are known to drive in female meiosis when a knob on chromosome 10 (Ab10) is present [62,63]. In spermatophytes like maize, female meiosis begins with a megasporocyte that undergoes two meiotic divisions, producing four haploid cells called megaspores in a linear tetrad (figure 4*b*) [64]. Similar to animal female meiosis, one of these haploid cells will contribute to the next generation; the others will degenerate. Importantly, it is the basal megaspore (the lower megaspore in the tetrad) that will produce the gametophyte [64,65]. When Ab10 is present in the genome, knobs containing a 180 bp repeat (knob180) act like ‘neocentromeres’ by moving towards spindle poles, though not by interaction with typical kinetochore proteins [66,67]. These ‘neocentromeres’ are able to move faster than the canonical centromeres and when combined with a crossover that produces heteromorphic dyads (figure 4*c*), knob chromatids are deposited in the upper and lower megaspores in the linear tetrad (figure 4*b*) [68,69]. Preferential segregation to the lower megaspore by means of faster ‘neocentromeres’ results in 83% transmission bias of knob domains (table 1) [32]. A recent 2018 study leveraging advances in next-generation sequencing (RNA-seq and PacBio) combined with traditional BAC sequencing revealed a gene cluster of eight or nine tandemly arrayed kinesin genes which the authors called *Kinesin driver* or *Kindr* [70]. Two previously observed Ab10 mutants in which drive does not occur were analysed for *Kindr* mutations and found to be epigenetically modified. These mutants silenced *Kindr* with small interfering RNAs or DNA methylation, revealing epigenetic methods for the suppression of drive. This is interesting because the rest of the genome is expected to experience selective pressure to suppress drive, and it is possible that this epigenetic control may represent a common route to suppression. Experimentally inhibiting *Kindr* with RNAi also led to the loss of drive and confirmed that *Kindr* is required for knob preferential segregation [70]. The authors were able to show that *Kindr* is a minus-end-directed kinesin-14 motor that interacts with the 180 bp repeats found in some knobs [70]. A second kinesin-14 gene which appears to be evolutionarily distinct (having an independent origin) from *Kindr* was later found on Ab10 as well [65]. This second kinesin motor has been called *TR-1 kinesin* (*Trkin*). Just as *Kindr* interacts with knob180 sequence and assists its preferential segregation, *Trkin* interacts with a minor tandem repeat called TR-1 and turns these repeats into ‘neocentromeres’. The authors suggest that the co-occurrence of knob180 and TR-1 repeats, combined with the fact that *Trkin* functions in prophase prior to *Kindr*, indicates *Trkin* as a secondary control of meiotic drive where the primary control is *Kindr* [65]. As meiotic drive is expected to invoke an evolutionary arms race between the driver and the rest of the genome, the implications of a secondary mechanism are very interesting.

The repeated use of kinesin and kinesin-related proteins is also of interest. While meiotic drive in maize relies on kinesin-14 motor proteins *Kindr* and *Trkin*, centromere drive in mice involves a kinesin-13 motor protein MCAK, though MCAK is not motile [71]. Additionally, kinesin-related genes have been found on B chromosomes (discussed below) in various species (*KIF11* in *Astatotilapia latifasciata*, *KIF20A* in *Eyprepocnemis plorans*, *KIF23* in *Apodemus peninsulae*, and *CENPE* in *Apodemus flavicollis*, *Metriaclima lombardoi* and *Astatotilapia latifasciata*) [72–75]. Furthermore, a chromokinesin *nod* is thought to be involved in female meiotic drive in *Drosophila melanogaster* [76]. It seems likely that kinesins and kinesin-related proteins are a common component of female meiotic drive.

Other non-centromeric loci that experience female meiotic drive, including homogeneously staining region (HSR) on chromosome 1, responder to drive 2 (R2d2) on chromosome 2 and ovum mutant (Om) on chromosome 11 in mice, are prime opportunities for characterization of non-centromeric meiotic driver in animals [33–35,77,78]. The underlying molecular mechanisms are currently unknown, and it is possible that they function like ‘neocentromeres’ the same way maize knobs do [34,70]. Some similarities exist between these four non-centromeric examples, including that they experience relatively high-transmission ratio distortion (83% for knobs, 85% for HSR and 95% for R2d2 – table 1), and drive has been shown to predominantly occur in meiosis II for all four systems [33,35,62] (F.E.C. & T.A. 2020, unpublished data). This preferential segregation in meiosis II is due to crossovers positioned between the centromere and the driving locus, resulting in heteromorphic dyads that require segregation of distinct chromatids in meiosis II (figure 4*c*) [35,79] (F.E.C. & T.A. 2020, unpublished data). It is interesting to note that the metaphase II spindle in mice is parallel to the cortex, as opposed to the perpendicular metaphase I spindle [80]. Since parallel spindles won't have the opportunity to establish spindle asymmetry through cortical signals, as discussed above, these non-centromeric meiotic drivers may be exploiting a different asymmetry to preferentially transmit to the next generation. Future studies are required to better understand the asymmetry in meiosis II and how recombination influences the success of non-centromeric drive systems.

## 3. Drive involving univalents

### 3.1. XO *Mus musculus*

In the context of Mendelian segregation, univalent (unpaired) chromosomes in meiosis I should segregate with equal frequency to the egg and first polar body (figure 1*b*). Instead, several examples in nature suggest univalent chromosomes can preferentially segregate, to the egg in some species and to the polar body in others. An early recognized example is that of XO female mice [81]. Studies from the 1970s first suggested that the univalent X chromosome preferentially segregated to the egg in meiosis I (depicted in figure 2*c*) [82,83]. Studies three decades later confirmed preferential segregation and also illuminated the more complex segregation patterns of the univalent X [28]. In *Mus musculus* mice, equational division of the univalent X chromosome, separating sister chromatids, did occur in meiosis I in some oocytes [27,28]. However, the X chromosome univalents that segregated intact experienced preferential segregation to the egg (table 1). LeMaire-Adkins & Hunt suggested that the underlying mechanism was related to differential ‘weight’ on either side of the spindle; where having more centromeres or larger chromosomes attached to one side creates a ‘heavier’ pole which mechanistically orients toward the oocyte centre and away from the cortex during spindle migration [28].

### 3.2. Trisomic and triploid *Caenorhabditis elegans*

**Figure 5.**
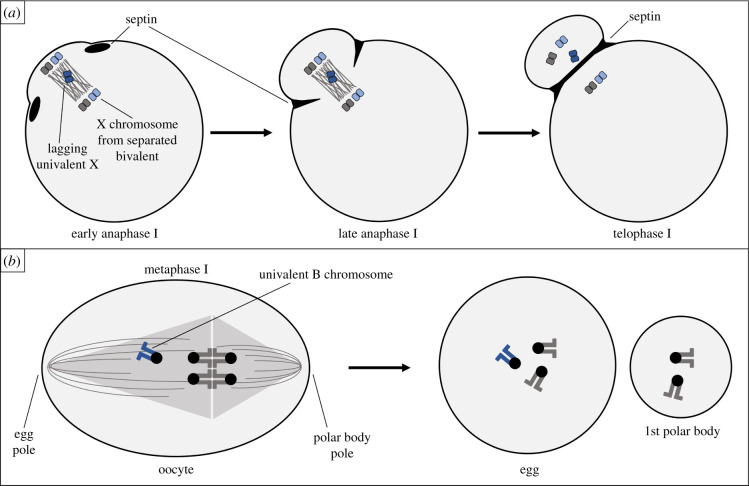
Univalent drive in worms and grasshoppers. (*a*) Univalent X chromosomes preferentially segregate to the polar body in *Caenorhabditis elegans*. These univalents lag in meiosis I and are captured by the septin tube during contractile ring activity. (*b*) Univalent B chromosomes in *Myrmeleotettix maculatus* are preferentially segregated to the egg. The side of the spindle facing the egg pole is longer than the side facing the polar body pole. Univalent B chromosomes are not aligned at the metaphase plate and can be randomly found anywhere along the spindle. The B chromosome is more likely to reside on the side of the spindle facing the egg pole due to this spatial asymmetry and is therefore more likely to be incorporated into the egg.

Another X chromosome observed to form univalents and experience preferential segregation is the X chromosome of *Caenorhabditis elegans* (figures 2*d* and 5*a*). In *C. elegans,* an XO genotype produces males, and an XX or XXX genotype produces hermaphrodites, which create both sperm and eggs [84]. In trisomy X worms, two X chromosomes form a bivalent while the third homologue is left to form a univalent in metaphase I [85]. These XXX worms produce an excess of haplo-X eggs compared to diplo-XX eggs, suggesting preferential segregation of the univalent X to the polar body during female meiosis. This preferential segregation is in the opposite direction of that observed in XO mice. We have introduced preferential segregation during female meiosis as a mechanism by which a selfish genetic element can achieve drive. So why would preferential segregation extrude a univalent chromosome with the polar body? Unlike the examples of trivalents discussed above where the metacentric chromosome could be the ‘winner’ if segregated to the egg, or the ‘loser’ if segregated to the polar body making the telocentric chromosomes the ‘winners’, univalent chromosomes have no paired chromosome that can become the ‘winner’ when preferential segregation directs the univalent to the polar body. As such, this is an example of preferential segregation, but not an example of female meiotic drive. However, we include this system as a relevant example of preferential segregation of univalent chromosomes because a better understanding of preferential segregation will lead to a better understanding of female meiotic drive. This will be further discussed below.

A 2015 study examined not only these XXX worms, but two mutants that each have a pairing defect, one specific for the X chromosome and the other specific for chromosome V [29]. In these mutants, the two X chromosomes and the two V chromosomes, respectively, form two univalents in metaphase I instead of one bivalent. In XXX individuals, the univalent X preferentially segregates to the polar body. In mutants with two univalent X chromosomes, there is a higher-than-expected frequency of eggs lacking both X chromosomes, indicating preferential segregation to the polar body of each univalent. Furthermore, chromosome V univalents in mutants also showed preferential segregation to the polar body, suggesting a general property of univalents to be preferentially extruded to the polar body in *C. elegans*. The 2015 study [29] also determined that, in these mutants, the univalent X chromosomes are bioriented at metaphase I and that univalent chromosomes in both types of mutants lag during anaphase I. Cortes *et al*. separated the lagging chromosomes into ‘early’ and ‘late’ resolving categories and made a strong case that ‘early’ resolving univalents preferentially segregate by stochastically attaching closer to one pole prior to spindle migration, followed by a bias of that side of the spindle to a position closer to the cortex. This is similar to the ‘heavier’ pole hypothesis put forward by LeMaire-Adkins & Hunt [28], where the side of the spindle with the additional chromosome is heavier and this extra weight affects its subsequent orientation. But again, it is in the opposite direction, as here the heavier pole would orient towards the polar body. The authors also demonstrate with a series of mutants that ‘late’ resolving univalents preferentially segregate via capture by the septin tube during contractile ring activity (figures 2*d* and 5*a*). They discovered contractile ring positioning displacement such that scission occurs between the univalent(s) and the egg chromosome mass (figures 2*d* and 5*a*) [29]. The fact that two distinct mechanisms could be resulting in the preferential segregation of univalent chromosomes in *C. elegans* is intriguing. Future studies could reveal if univalent X chromosomes in XO female mice similarly lag in anaphase.

A more recent study examined preferential segregation in triploid *C. elegans* Vargas *et al*. [86] found that the univalent third homologue of each chromosome type preferentially segregated to the polar body such that triploid hermaphrodites produced more euploid offspring than expected if univalents segregated at random. Another notable example is that of triploid female oysters, which, when mated to diploid males, produce mostly diploid offspring [26].

### 3.3. Could preferential segregation of univalents have evolved under natural selection?

The argument has been put forth for XO mice, XXX worms and triploids (worms and oysters) that the preferential segregation is evolutionarily advantageous as it selects for a return to a euploid state [26,28,29,86]. Essentially, preferential segregation toward the egg in XO mice leads to more XX daughters and preferential segregation towards the polar body in XXX worms leads to more XX progeny. The suggestion of evolutionary pressures selecting for preferential segregation, but in opposite directions, might first raise the question of whether evolutionary biologists are simply looking for signatures of adaptation, even in what could be described as a simple mechanical byproduct. However, we think it is worth pointing out that *C. elegans* are better served by preferential segregation towards the polar body. In general (in no specific taxa), XO and XXX are aneuploid genotypes that exhibit univalent X chromosomes during meiosis. Preferential segregation in an XO female would produce XX offspring if the univalent segregates to the egg, while preferential segregation in an XXX female would produce XX offspring if the univalent segregates to the polar body. But in *C. elegans*, XO individuals are not female (or rather hermaphrodites), they are male, and in males, meiosis is symmetric. So, there is only one aneuploid X chromosome genotype in *C. elegans* that can be ‘corrected’ by preferential segregation of univalents in female meiosis. Furthermore, this direction of preferential segregation also allows a return to the euploid state in triploid worms [86]. Comparatively, triploid mice are inviable, and female mice can be XO or XXX, though only XO females are fertile [87,88]. The observed direction of preferential segregation of univalents in mice could suggest a bigger benefit to resolving XO aneuploidy rather than XXX aneuploidy, possibly due to other associated fitness costs of XXX individuals.

In their 2000 study, LeMaire-Adkins & Hunt [28] suggested that the preferential segregation of univalent X chromosomes in mice was not a ‘real’ case of ‘genetically controlled meiotic drive’, because neither the genetic background nor the specific X chromosome appeared to determine the efficiency of drive. Rather, it seemed that preferential segregation of the univalent was the product of some aspect of asymmetric female meiosis, specifically the spindle [28]. This line of reasoning is further strengthened by preferential segregation of univalents in *C. elegans* that is clearly not a case of meiotic drive at all. Considering the hypothesis put forth by many that univalent preferential segregation is evolutionarily advantageous, it seems reasonable that natural selection may have shaped some aspect of female meiosis (e.g. the spindle) to intrinsically sort unpaired chromosomes towards either the egg or polar body [26,28,29,86]. If this aspect of female meiosis (or aspects, as Cortes *et al*. suggested two distinct mechanisms) is similarly controlling univalent preferential segregation across the three examples above (XO mice, XXX worms and triploid worms/oysters), perhaps that plays a role in the repeated observation among univalents of an approximate 2 : 1 transmission bias (table 1). Furthermore, if this preferential segregation of univalents (in either direction) is even somewhat universal, it could be another, underappreciated opportunity for selfish genetic elements.

### 3.4. B chromosomes

B chromosomes are another type of chromosome known to frequently be univalent and to experience female meiotic drive [4,89,90]. In some species, additional unique chromosomes (i.e. not a case of triploidy, trisomy or other aneuploidy) are found in some individuals but not others and are called B chromosomes [91]. They are thought to originate from the standard or ‘A’ chromosomes, but experience different selective pressures as they are non-essential to the organism and are frequently selfish [5,72,92,93]. From species to species, B chromosomes vary in size, sequence and behaviour. They have been found in hundreds of species of plants, animals and fungi, but are associated with surprisingly few phenotypes [3,92–94]. B chromosomes can vary in number between individuals and between cells of an individual, often producing univalents during meiosis I [4]. The frequency of a B chromosome among members of a population is the result of a combination of three factors: the strength of drive, the suppression of drive by the rest of the genome and any fitness effects imposed by the B chromosome [4,5,90]. B chromosomes are known to drive in many ways (not just female meiotic drive) and can accumulate by increasing in number per individual or as an increase in the frequency of individuals that carry a B chromosome in the population [4,90,92]. The tried-and-true example of B chromosome meiotic drive has long been the grasshopper, *Myrmeleotettix maculatus* (figure 5*b*), though the first described case was the B chromosome in lilies, *Lilium callosum* [95–97]. In both species, the B chromosomes are positioned off-centre from the metaphase plate during metaphase I (figure 5*b*) [3]. Also common to both species is a spindle asymmetry, where the side of the spindle facing what will become the egg is longer than the side that will be excluded in the polar body (figure 5*b*). Assuming the B chromosomes are located randomly on the spindle, it is more likely that the B chromosomes will be located on the longer egg side of the spindle and be incorporated into the egg. B chromosomes are also thought to experience female meiotic drive in several plant species (including herd's grass, hawkweed and the Sitka spruce), several animal species (including mealy bugs, rats, lemmings and fish), and at least one fungus, *Zymoseptoria tritici* [92,98–101]. While the field of B chromosome research has seen a recent surge in genetic, genomic, proteomic and cytogenetic studies leading to several exciting discoveries about the molecular nature of B chromosome drive, these discoveries involve species with B chromosomes that accomplish drive through means other than preferential segregation in female meiosis. Unfortunately, little advancement has been made in our understanding of how B chromosomes preferentially segregate in female meiosis, largely due to the difficulty in analysing oogenesis compared to spermatogenesis in many of these species. Recent genomic studies have, however, revealed an enrichment of meiosis and chromosome segregation-related genes on B chromosomes in several different species [96,98,102–106]. This recurrent enrichment suggests these B-located gene duplications are functional and assist in the B chromosome's meiotic drive, perhaps by overriding checkpoints in order to proceed through meiosis, despite deficiencies in pairing and alignment, or by altering the timing of anaphase onset (earlier or later) in order to skew segregation. It is also possible and has been suggested that B chromosomes achieve drive in female meiosis through a more passive mechanism [4,90,107]. As many B chromosomes have been shown to form univalents in meiosis, even when present in even numbers, it is possible that B chromosomes preferentially segregate in female meiosis through a similar mechanism as univalent chromosomes in mice and worms. In other words, B chromosomes could acquire the ability to drive simply by taking advantage of an oocytes pre-existing tendency to preferentially segregate univalent chromosomes towards the egg. If this is true, B chromosomes would be expected to drive via female meiosis (as opposed to other cell divisions) only in species that intrinsically segregate univalents towards the egg (i.e. mice but not worms). Conversely, B chromosomes would be expected to drag (transmit below Mendelian expectation) in female meiosis, and/or increase their transmission through means other than meiotic drive, in species that segregate univalents towards the polar body.

### 3.5. Do germline restricted chromosomes experience female meiotic drive?

A very similar type of chromosome, called the germline restricted chromosome (GRC), is found in every individual of every species of songbird examined to date and is hypothesized to experience female meiotic drive [5,108–112]. This GRC is present in the germline of both males and females, but entirely absent from somatic cells [109,111]. Most females (approx. 90%) have two GRC while males have been found to carry a single GRC, which is lost during spermatogenesis [108,109,113]. There is yet to be a definitive answer for the inheritance and maintenance of the GRC as meiotic divisions are difficult to study in birds [112]. Interestingly, the GRC has been suggested to have evolved from a B chromosome [5,110,112]. It is thought that the elimination through males and preferential segregation in females effectively ‘stabilized’ the B chromosome among populations, while elimination from somatic cells nullified associated fitness costs, allowing the chromosome to gain a more ‘symbiotic’ role in the germline [5,110,112,114]. If this is true, and if the GRC does use preferential segregation in meiosis, it may be the only known example where female meiotic drive has been incorporated into the life cycle of the organism rather than selectively suppressed by the host genome. A lack of methods to examine female meiosis in these species is currently an obstacle to confirming the involvement of female meiotic drive. But should the next decade see progress in this area, better characterization of the GRC would provide many evolutionarily and mechanistically important insights into the role of female meiotic drive.

## 4. Concluding remarks

We have entered an era where female meiotic drive is recognized as both pervasive across taxonomic groups and evolutionarily relevant. For this reason, recent years have seen marked progress in our understanding of the molecular mechanisms contributing to female meiotic drive, in multiple systems. In this review, we've categorized these drive systems not just as centromeric or non-centromeric, but also by whether the chromosome benefiting from drive forms univalents, bivalents or trivalents in meiosis I. We've discussed how drive involving bivalents and trivalents are similarly controlled by centromeres. This means that in addition to the rapid evolution of centromere DNA and proteins hypothesized by Henikoff *et al.* [23] and Malik *et al.* [24], centromere drive is also responsible for rapid karyotype evolution [16,24,37]. As centromere drive in mice has been shown to require the recruitment of destabilizing factors, the pericentromere has an underappreciated role in this form of drive. Furthermore, we highlighted a common feature among non-centromeric systems, a strong drive during meiosis II. Finally, we've suggested that preferential segregation involving univalent chromosomes may be mechanistically similar across systems. As these univalents can be preferentially segregated to the egg or polar body, depending on the species, it is possible that this represents a passive mechanism of preferential segregation dependent on some meiotic feature that evolved under natural selection to favour a return to euploidy.

We expect the next decade will bring equally exciting progress in this field. Here, we briefly discuss key areas that require future attention. In order to understand how prevalent the mechanisms described above are, it would be highly beneficial for similar studies to characterize drive during oogenesis in a variety of organisms across major taxonomic groups. Unfortunately, the biggest road block to increasing taxonomic sampling is the general difficulty in examining female meiosis, a problem seen in many species. While methods for doing so in a few key model species offer a thoroughly established and versatile toolset, further development of methods which work for a larger range of species would undoubtedly have a massive impact on this field. For those systems and species that have been examined to date, a continued effort to fully describe female meiotic drive would benefit from mechanistically connecting the molecular changes introduced by a given selfish genetic element to the structural asymmetry being manipulated. It is worthwhile to note that much of the work describing the molecular components of drive focuses on meiosis I, though drive can occur during meiosis II as well. Of particular interest is a comparison of mechanisms between meiosis I and II, and whether or not a single selfish genetic element could manipulate both meiosis I and II through the same mechanism. Furthermore, if a non-centromeric drive allele can only achieve drive during meiosis I or during meiosis II, then control of recombination can result in either heteromorphic or homomorphic dyads (balanced or unbalanced bivalent, respectively), altering the opportunity for drive. As an example, if an allele can manipulate oogenesis and drive during meiosis I only, then recombination that produces heteromorphic dyads (a balanced bivalent) would prevent drive during meiosis I. For this reason, a selfish genetic element might evolve a means to reduce recombination, or recombination might even be incorporated into a suppression mechanism meant to decrease drive. A measurement of recombination rates across chromosomes in species experiencing female meiotic drive could reveal such a phenomenon. Among centromeric drive systems, the pericentromere should be investigated as a potential contributor to altered transmission rates. If pericentromeres are routinely involved in centromere drive, it would be interesting to quantify how frequently the pericentromere increases, and benefits from, drive versus how frequently it suppresses drive. Lastly, as the components of these drive mechanisms are identified, the genes involved should be analysed for repetitive use. If the same genes or gene families are incorporated into drive mechanisms in multiple systems, it would suggest that those genes are vulnerable to exploitation by selfish genetic elements.

## Data accessibility

This article has no additional data.
